# Exploring status and determinants of prenatal and postnatal visits in western China: in the background of the new health system reform

**DOI:** 10.1186/s12889-017-4601-4

**Published:** 2017-07-20

**Authors:** Xiaojing Fan, Zhongliang Zhou, Shaonong Dang, Yongjian Xu, Jianmin Gao, Zhiying Zhou, Min Su, Dan Wang, Gang Chen

**Affiliations:** 1 School of Public Health, Center of Medical Science, Xi’an Jiaotong University, Xi’an,Shaanxi, People’s Republic of China; 2 School of Public Policy and Administration, Xi’an Jiaotong University, 76 Yanta West Road, Xi’an, Shaanxi, People’s Republic of China; 3 School of Medicine, Flinders University, Adelaide, Australia

**Keywords:** Prenatal visits, Postnatal visits, Inequality, Health system reform

## Abstract

**Background:**

Prenatal and postnatal visits are two effective interventions for protection and promotion of maternal health by reducing maternal mortality and improving the quality of birth. There is limited nationally representative data regarding the changes of prenatal and postnatal visits since the latest health system reform initiated in 2009 in Shaanxi, China. The aim of this study was to explore the current status and determinants of prenatal and postnatal visits in the background of new health system reform.

**Methods:**

Data were drawn from two waves of National Health Service Surveys in Shaanxi Province which were conducted prior and post the health system reform in 2008 and 2013, respectively. A concentration index was employed to measure the degree of income-related inequality of maternal health services utilization. Multilevel mix-effects logistic regressions were applied to study the factors associated with prenatal and postnatal visits.

**Results:**

The study sample consists of 2398 women aged 15-49 years old. The data of the 5th National Health Services Survey in 2013 showed in the criterion of the World Health Organization (WHO), the percentage of women receiving ≥4 prenatal visits was 84.79% for urban women and 82.20% for rural women, with women receiving ≥3 postnatal visits were 26.48 and 25.29% for urban and rural women respectively. In the criterion of China’s ≥ 5 prenatal visits the percentages were 72.25% for urban women and 70.33% for rural women; 61.69% of urban women and 71.50% of rural women received ≥1 postnatal visits. As for urban women, the concentration index of postnatal visit utilization was −0.075 (95% CI:-0.148, −0.020) after the health system reform. The determinants related to prenatal and postnatal visits were the change of reform, women’s education, parity and the delivery institution.

**Conclusions:**

This study showed the utilization of prenatal and postnatal visits met the requirement of the WHO, higher than other areas in China and other developing countries after the new health system reform. The new health system reform increased the utilization of postnatal visits in poor urban women and improved the frequency of prenatal and postnatal visits in rural women.

**Electronic supplementary material:**

The online version of this article (doi:10.1186/s12889-017-4601-4) contains supplementary material, which is available to authorized users.

## Background

Maternal health refers to the health of women during pregnancy, childbirth and the postpartum period. Although it is often a positive and fulfilling experience, for many women in developing countries, motherhood is associated with suffering, ill-health and even death. Maternal mortality rate (MMR) is an important indicator to evaluate the health status in developing countries and it has a strong association with the utilization of maternal health services [1]. The process in maternal mortality ratio had been slow recently [2], but in developing countries it is still approximately 200 times higher than that in Western countries [3]. In China, the MMR decreased from 56.2/100,000 in 1998 to 23.2/100,000 in 2013 [4, 5], still higher than England, Sweden, and the United States (around 10 per 100,000) [6].

Prenatal and postnatal visits are two effective approaches of maternal health services to detect and save the life of mother and newborn from life threatening complications (such as primary and secondary postpartum hemorrhage, infection, injuries, and hypothermia) and to ensure the healthy outcome for both mother and newborn [7]. The World Health Organization (WHO) recommends that the prenatal visits should include at least four visits to a health facility during an uncomplicated pregnancy [8], whereas at least three postnatal care visits are recommended for all mothers and newborns [9]; however these criteria have only been partially met in developing countries. In Africa, only 44% of pregnant women attended prenatal visits at least four times [10], in Indonesia 77.9% women received at least four prenatal visits [11]. The postnatal period is the time from delivery of the baby till the first 42 days [12]. Only 24% of women in Bangladesh and 25% in Nepal received postnatal care [13, 14].

In China, the measures for the administration of the national maternal system proposed prenatal visits should not be less than five times, whilst at least 70% of urban women and 60% of rural women should receive three postnatal visits at home by health-care providers [15]. According to a representative survey, in 1971-2003 the percentage of more than five prenatal visits was lower than 60% in China [16]. More recently, a cross-sectional survey conducted in 2007–2009 found that only 43.8% of rural women received five or more prenatal visits in Shaanxi Province, highlighting an urgent issue for pregnant women in rural China [17]. With regard to the postnatal visits, data from the 3rd National Health Service Survey (NHSS) in 2003 showed that the rate of women having at least one postnatal visit was 60% in the urban areas, 52% in the rural areas and 37% in the poorest rural areas [18]. A survey in Chongqing found that the percentage of women having one postnatal visit was 70.2%, and three postnatal visits was only 28.9% in 2008 [19].

The underutilization of maternal health services is generally related to unavailability, inaccessibility and the poor quality of health services which was common in developing countries [20, 21]. China has initiated a new health system reform since 2009, with one of the key goals aiming to promote the gradual equalization of basic public health services and narrowing the gap in access to those services that exists between rural and urban areas, including the maternal health services in the nation [22, 23]. The reform of the public health services recommends that maternal women should receive more than five prenatal visits and at least one postnatal visit. Therefore, the utilization of prenatal and postnatal visits should be improved after the new health system reform. So far there has been no empirical evidence to show whether or not the health system reform has improved the maternal health services utilization, and whether the health reform has reduced the inequalities of utilization. This study aims to fill this gap using two national representative surveys conducted in Shaanxi Province, prior and post health system reform.

## Methods

### Data source

Data were drawn from two waves of National Health Service Surveys (NHSS) conducted in Shaanxi Province. The NHSS is a national representative survey commissioned by the National Health and Family Planning Commission of China. In each province, a multistage stratified cluster random sampling method was used to ensure the samples be representative of the whole population of each province. Shaanxi Province is located in the northwest of China, with a population of around 37 million. The new health system reform was initiated in 2009 and added the basic public health services to urban and rural residents. The NHSS were conducted before the health system reform in June of 2008 and after the health system reform in September of 2013, respectively. During the survey, all household members were interviewed individually using a structured household questionnaire (Additional files 1 and 2). In total, data from 660 women in 2008 and 1738 women in 2013 were utilized for this analysis.

Considering the fact that residents living in rural areas are more likely to encounter barriers (inadequate health care insurance coverage, long distances to health care facilities, lack of transportation and an undersupply of particularly specialists) to receiving needed health care than people living in urban areas [24, 25], the rural sample and urban sample were analyzed separately.

### Statistical analysis

#### Prenatal and postnatal visits

The WHO recommends women aged 15-49 attend at least four prenatal visits in a health facility during an uncomplicated pregnancy [8]. The administration of the China National Maternal and Child Health Surveillance System proposed that prenatal visits should not be less than five times. In this study, we categorized ≤4 prenatal visits as unqualified prenatal visits and ≥5 as qualified prenatal visits. The WHO recommended at least three additional postnatal visits are required for mothers and newborns, on day 3 (48–72 h), between days 7–14 after birth, and 6 weeks after birth. In the NHSS data, we observed that a very low percentage of women had attained the recommended number of postnatal visits (9.1% for urban women and 5.4% for rural women). This may be because that the new maternal health system reform has added the postnatal visits to the essential public health service from 2009 so we generated a dummy variable to indicate whether women had any postnatal visits.

#### Estimate of concentration index

A concentration index is employed to measure the degree of income-related inequality of healthcare utilization and ranges from −1 to +1 [26, 27]. A positive concentration index means that high-income people utilize more maternal health services than their low-income counterparts. Meanwhile, the concentration index is negative if the low-income group utilizes more maternal health services than their rich counterparts [28]. In extreme cases, the concentration index reaches −1 if all healthcare resources are utilized by the poor whereas the index is +1 if the rich are favored in healthcare utilization. Healthcare is equitably utilized by the poor and the rich when the index is 0. The formula for computing the concentration index is:

1 $$ C=\frac{2}{\mu}\mathit{\operatorname{cov}}\left({y}_i,{R}_i\right) $$

where C stands for concentration index, *y*_*i*_ is prenatal or postnatal visits index,  *μ* is the mean of prenatal or postnatal visit index, and *R*_*i*_ is the fractional rank of annual personal consumption expenditure distribution.

#### Multilevel mixed-effects model

A multilevel mixed-effects generalized linear mixed model has been adopted in this study, taking into account the hierarchical survey data structure [29]. This is an extension of linear mixed models to allow response variables from different distributions (such as binary responses) and includes both fixed and random effects. The form of the multilevel mix-effects generalized linear model is:

2 $$ \mathrm{g}\left\{ E\right.\left.\left( y| X, u\right)\right\}=\mathrm{X}\beta +\mathrm{Z} u,\kern0.5em \mathrm{y}\sim \mathrm{F} $$

where *y* is the n × 1 vector of responses from the distributional family F, *X* is an n × p covariate matrix for the fixed effects *β*, and Z is the n × q covariate matrix for the random effects *u*. The *Xβ* + Z*u* part is called the linear predictor. If *y*is distributed as Bernoulli (binary responses), we have mixed-effects logistic regression which was used in this study. The Eq. (2) can be written as:

3 $$ \mathrm{logit}\left\{ E\right.\left.(y)\right\}=\mathrm{X}\beta +\mathrm{Z} u,\kern0.5em \mathrm{y}\sim \mathrm{Bernoulli} $$

Maternal health service utilization is a multifaceted phenomenon that is a function of numerous factors including household income, health insurance status, health care user’s attitudes and beliefs, availability of health services, marital status, and maternal education [30–34], based on the previous literature and the characteristics of the questionnaire, we selected some variables for exploratory analysis on association between them and prenatal and postnatal visits. In this study, the fixed effect were maternal age (≤25, 26-30, ≥31), education (≤Primary school, Middle school, ≥high school), employment (whether employed during the survey),annual Household income (five equal quintiles: the poorest, poorer, medium, richer, and the richest), delivery institution (Secondary and above, ≤Primary institution), delivery approach (Vaginal, Caesarean), parity (≤1, ≥2), and weight of newborn (Low birthweight, Normal birthweight); the random effect were the individuals and their households. Specifically, one family respondent who is familiar with the household financial situation was asked to report the total annual household income and consumption expenditure for the past year in the NHSS survey. The total consumption expenditure can be divided into eight parts: health care, food, daily living, transportation and communication, housing, education, entertainment, and others. Owing to the potential under-reporting issue, the annual household income was proxy by total annual household consumption expenditure minus household health expenditure following the literature [35, 36]. Odds risk (OR), together with 95% confidence intervals (CIs), were used to evaluate the association of the occurrence of qualified prenatal and postnatal visits with selected factors. All analyses were used STATA statistical software version 12.0. A two-tailed *P* value <0.05 was considered statistically significant.

### Quality control

In order to ensure the results of the survey to be accurate and reliable, survey supervisors revisited 5% of the sampled households to check the accuracy of data recorded by interviewers. In this process, 14 key questions were asked again to check the consistency of the information recorded. The consistency rates of the key questions recorded between the first and second visits was over 95%. The Myer’s Blended index was 1.67 in 2008 and 1.62 in 2013, indicating that in both years there was no significant difference between the sampled age distribution and the overall age distribution of Shaanxi Province [37].

## Results

The characteristics of the participants by urban-rural region are presented in Table 1. The mean age of the participants were 28.67 years old, the distribution of age between urban women and rural women were statistically different (χ^2^ = 34.8, *P* < 0.001). Compared to women in rural regions, women from urban regions were significantly more likely to receive higher levels of education, have higher household income, or deliver in secondary institutions and above (all *P* < 0.001). On the other hand, women from urban regions were significantly less likely to be employed during the survey, adopt vaginal delivery, or have more than two parities, as compared to women in rural regions (all *P* < 0.001). No statistically significant difference was observed for the weight of the newborn (*P* > 0.05). In terms of health reform, there are significant differences prior and post the health system reform regarding household income, delivery institution and way, parity and weight of newborn (all *P* < 0.05).

**Table 1:** Characteristics of participants [$\overset{-}{x}\pm s$/n (%)]

	*n*	Urban		Rural	
		Before reform	After reform	Before reform	After reform
Age (years)					
	28.67 ± 5.48	28.37 ± 4.24	28.60 ± 5.19	28.59 ± 5.69	28.82 ± 5.81
≤ 25	751	57(25.56)	195(27.46)	149(34.10)	350(34.05)
26–30	878	107(47.98)	302(42.54)	137(31.35)	332(32.30)
≥ 31	769	59(26.46)	213(30.00)	151(34.55)	346(33.65)
Education					
≤ Primary school	451	8(3.59)	78(11.02)	133(30.57)	232(22.59)
Middle school	1318	84(37.67)	371(52.40)	243(55.86)	620(60.37)
≥ High school	624	131(58.74)	259(36.58)	59(13.56)	175(17.04)
Employment					
No	532	95(43.38)	218(30.79)	372(85.32)	872(84.91)
Yes	1858	124(56.62)	490(69.21)	64(14.68)	155(15.09)
Annual Household income (Ren Min Bi)					
	22,402 ± 18,828	16,718 ± 9723	32,841 ± 24,662	9985 ± 7107	21,675 ± 14,717
Poorest	479	99(44.80)	87(12.27)	224(51.49)	69(6.71)
Poorer	479	54(24.43)	132(18.62)	108(24.83)	185(18.00)
Media	478	40(18.10)	146(20.59)	58(13.33)	234(22.76)
Richer	479	22(9.95)	164(23.13)	22(5.06)	271(26.36)
Richest	478	6(2.71)	180(25.39)	23(5.29)	269(26.17)
Delivery institution					
Secondary and above	1948	144(64.57)	661(93.10)	201(46.00)	942(91.63)
≤ Primary institution	450	79(35.43)	49(6.90)	236(54.00)	86(8.37)
Delivery way					
Vaginal	1673	156(98.73)	434(61.47)	329(95.64)	754(73.42)
Caesarean	562	2(1.27)	272(38.53)	15(4.36)	273(26.58)
Parity					
≤ 1	1381	193(86.94)	433(60.99)	258(59.58)	497(48.44)
≥ 2	1010	29(13.06)	277(39.01)	175(40.42)	529(51.56)
Weight of newborn					
Low birthweight	100	2(0.90)	32(4.51)	9(2.06)	57(5.54)
Normal birthweight	2298	221(99.10)	678(95.49)	428(97.94)	971(94.46)

### Comparisons on prenatal and postnatal visits before and after health reform

**Fig. 1:**
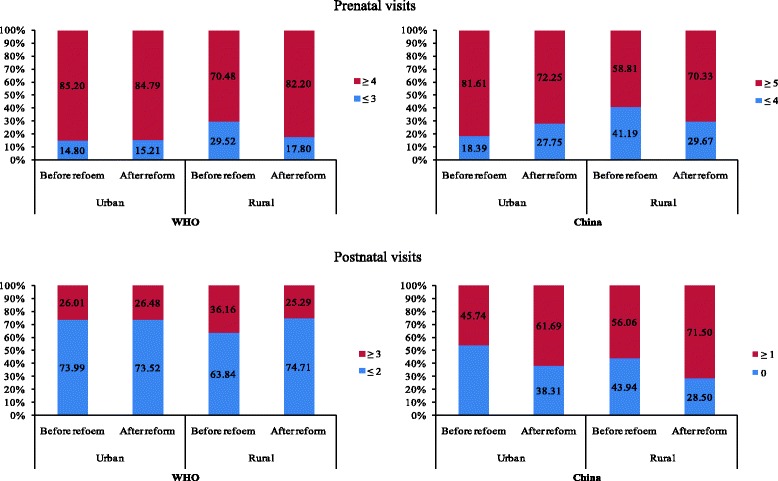
Comparisons on prenatal and postnatal visits before and after health reform

Comparisons on prenatal and postnatal visits prior and post the health system reform are presented in Fig. 1. For urban women, there were no statistically significant differences on changes of prenatal (85.20% vs. 84.79%, χ^2^ = 0.023, *P* = 0.881) or postnatal (26.01% vs. 26.48%, χ^2^ = 0.019, *P* = 0.890) visits according to the WHO criterion. According to China’s criterion, there was a drop in prenatal visits (χ^2^ = 7.826, *P* = 0.005) and an increase in postnatal visits after the health system reform (χ^2^ = 17.709, *P* < 0.001). For rural women, a significantly higher proportion achieved the recommended number of prenatal visits by both WHO and China criteria after health system reform (WHO: χ^2^ = 25.121, *P* < 0.001; In China: χ^2^ = 18.378, *P* < 0.001). As for the postnatal visits, after health system reform, the percentage of rural women achieving the WHO recommendation significantly decreased (36.16% vs. 25.29%, χ^2^ = 17.748, *P* < 0.001), whilst the percentage significantly increased according to the recommendation from the Chinese government (56.06% vs. 71.50%, χ^2^ = 32.984, *P* < 0.001).

### Inequality of prenatal and postnatal visits

Table 2 shows the concentration index of prenatal visits and postnatal visits, respectively. For urban women, the concentration index of prenatal visits decreased significantly from 0.053 (95% CI: 0.011, 0.093) before reform to 0.0004 (95% CI: -0.024, 0.025) post health system reform (*P* = 0.030). The concentration index of postnatal visits also changed significantly from 0.083 (95% CI: -0.019, 0.180) before reform to −0.075 (95% CI: -0.148, −0.020) post health system reform, indicating the postnatal visits were more used by the poorer women (*P* = 0.006). For rural women, there was an increasing trend for the concentration index of prenatal visits (*P* = 0.471), from 0.017 (95% CI: -0.012, 0.471) before reform to 0.029 (95% CI: 0.013, 0.045) post health system reform. The concentration index of postnatal visits remains indifferent (*P* = 0.080): 0.041 (95% CI: -0.017, 0.098) before reform and −0.017 (95% CI: -0.047, 0.012) after health system reform.

**Table 2:** Concentration index of prenatal and postnatal visits before and after health reform

	Before health reform			After health reform			*P*
	Concentration index	95% *CI*		Concentration index	95% *CI*		
		Lower	Upper		Lower	Upper	
Prenatal visits							
Urban	0.053	0.011	0.093	0.0004	−0.024	0.025	0.030
Rural	0.017	−0.012	0.471	0.029	0.013	0.045	0.471
Postnatal visits							
Urban	0.083	−0.019	0.180	−0.075	−0.148	−0.020	0.006
Rural	0.041	−0.017	0.098	−0.017	−0.047	0.012	0.080

### Factors associated with prenatal visits

Table 3 presents regression estimates on factors associated with prenatal visits for urban and rural women separately. For urban women, educations and household income were two significant factors. A positive association was evident for higher education level and prenatal visits. Compared with urban women educated ≤ primary school, the odds ratio of qualified prenatal visits (which were more than five times) was almost four times higher (OR = 3.97, 95% CI:1.25 ~ 12.57; *P* = 0.019). Regarding to the income level, urban women from the richest household were significantly fewer risk of having qualified prenatal visits as compared to the women from the poorest household (OR = 0.29, 95% CI: 0.09 ~ 0.96; *P* = 0.043).

**Table 3:** Multilevel mix-effects logistic regression of prenatal visits among urban and rural women

	Urban				Rural			
	*OR*	95% *CI*		*P*	*OR*	95% C*I*		*P*
Health reform								
Before	1.00				1.00			
After	0.81	0.39	1.68	0.573	1.97	1.13	3.44	0.018
Age (years)								
≤ 25	1.00				1.00			
26–30	1.41	0.72	2.77	0.313	1.05	0.61	1.82	0.858
≥ 31	0.84	0.39	1.81	0.664	1.37	0.72	2.60	0.331
Education								
≤ Primary school	1.00				1.00			
Middle school	2.50	0.95	6.56	0.063	3.13	1.74	5.64	<0.001
≥ High school	3.97	1.25	12.57	0.019	5.07	2.14	12.03	<0.001
Employment								
No	1.00				1.00			
Yes	1.12	0.65	1.95	0.683	0.62	0.34	1.15	0.131
Annual Household income								
Poorest	1.00				1.00			
Poorer	0.52	0.16	1.65	0.266	1.33	0.72	2.43	0.359
Medium	0.77	0.26	2.33	0.646	1.88	0.94	3.73	0.073
Richer	0.49	0.16	1.52	0.219	1.89	0.91	3.93	0.087
Richest	0.29	0.09	0.96	0.043	2.04	0.89	4.71	0.094
Parity								
≤ 1	1.00				1.00			
≥ 2	0.54	0.26	1.09	0.086	0.51	0.29	0.90	0.021

As for rural women, the odds ratio of qualified prenatal visits after the health system reform was approximately twice higher than those women before the health system reform (OR = 1.97, 95% CI:1.13 ~ 3.44; *P* = 0.018). There was a trend of increasing ratio of qualified prenatal visits with the increase of education, and the odds ratio of qualified prenatal visits in the education of middle school was three times higher than ≤primary school (OR = 3.13, 95% CI: 1.74 ~ 5.64; *P* < 0.001), and five times higher in ≥High school (OR = 5.07, 95% CI: 2.14 ~ 12.03; *P* < 0.001). A negative association was observed between the parity and prenatal visits. Rural women with parity ≥2 were in a lower ratio of qualified prenatal visits than those women with parity ≤1 (OR = 0.51, 95% CI: 0.29 ~ 0.90; *P* = 0.021).

### Factors associated with postnatal visits

Table 4 presented regression estimates on factors associated with postnatal visits. For urban women, the odds ratio of qualified postnatal visits (which were more than once) post reform was almost seven times more than before the reform (OR = 6.75, 95% CI:1.93 ~ 23.63; *P* = 0.003). Compared with the secondary and above delivery institutions, the odds ratio of qualified postnatal visits of women who were delivered in ≤primary institution were about four times higher (OR = 4.12, 95% CI:1.38 ~ 12.29; *P* = 0.011). Meanwhile, the odds ratio of qualified postnatal visits in rural women after the health system reform was almost 3.2 times higher than those women before the health system reform(OR = 3.15, 95% CI:1.54 ~ 6.48; *P* = 0.002). The odds ratio of qualified prenatal visits in the education of middle school was 2.2 times higher than ≤ primary school (OR = 2.16, 95% CI: 1.23 ~ 3.79; *P* = 0.008).

**Table 4:** Multilevel mix-effects logistic regression of postnatal visits among urban and rural women

	Urban				Rural			
	*OR*	95% *CI*		*P*	*OR*	*9*5% *CI*		*P*
Health reform								
Before	1.00				1.00			
After	6.75	1.93	23.63	0.003	3.15	1.54	6.48	0.002
Age								
≤ 25	1.00				1.00			
26–30	1.12	0.63	2.00	0.698	1.07	0.62	1.86	0.795
≥ 31	0.58	0.28	1.21	0.145	1.87	0.95	3.68	0.069
Education								
≤ Primary school	1.00				1.00			
Middle school	1.13	0.50	2.54	0.764	2.16	1.23	3.79	0.008
≥ High school	1.46	0.59	3.61	0.407	2.02	0.94	4.68	0.070
Employment								
No	1.00				1.00			
Yes	1.69	0.96	2.97	0.067	1.70	0.92	3.12	0.089
Annual Household income								
Poorest	1.00				1.00			
Poorer	1.02	0.38	2.74	0.969	1.20	0.64	2.26	0.574
Medium	0.74	0.28	1.93	0.533	1.61	0.80	3.22	0.182
Richer	0.77	0.29	2.01	0.593	1.36	0.66	2.78	0.403
Richest	0.55	0.20	1.50	0.243	1.24	0.55	2.80	0.598
Parity								
≤ 1	1.00				1.00			
≥ 2	1.01	0.56	1.83	0.961	0.79	0.45	1.38	0.406
Delivery institution								
Secondary and above	1.00				1.00			
≤ Primary institution	4.12	1.38	12.29	0.011	1.28	0.68	2.39	0.442
Delivery way								
Vaginal	1.00				1.00			
Caesarean	0.79	0.47	1.35	0.390	1.03	0.60	1.78	0.916
Weight of newborn								
Low birthweight	1.00				1.00			
Normal birthweight	0.35	0.09	1.37	0.132	0.78	0.28	2.22	0.647

## Discussion

By using the Shaanxi Province data of the 4th and 5th National Health Services Survey, this paper empirically studied the influence of health system reform on the maternal health services utilization of prenatal and postnatal visits among urban and rural women in Shaanxi Province, Western China. The data of the 5th National Health Services Survey in 2013 showed 84.79% of urban women and 82.20% of rural women received the WHO recommended minimum number of prenatal visits, which was more than the WHO recommended percent of 80% [38], and higher than the visits in Africa (44%) [10] and Indonesia (78%) [11]. In the criterion of China, the percentages (urban: 72.25%; rural: 70.33%) were also higher than the investigation in 1971–2003 (60%) in China [16] and 2007–2009 [17] in Shaanxi (43.8%), even higher than the result of 52.9% in Western China in 2011 [39]. From the point of postnatal visits, 26.48% of urban women and 25.29% of rural women received a similar percentage to Chongqing (28.9%) which the WHO recommended (≥3 postnatal visits) [19]. 61.69% of urban women and 71.50% of rural women received ≥1 postnatal visits, which was 3 to 4 times than the rate in Bangladesh (24%) [13] and Nepal (25%) [14], and a little higher than the 3rd National Health Service Survey in 2003 in the urban areas (60%) and rural areas (52%). Therefore, the status utilization of prenatal and postnatal visits met the requirement of WHO, and were higher than other areas in China and other developing countries.

China’s new health system reform aims to promote equal basic public health services, allowing urban and rural residents to have the same opportunity to receive essential public health services, including the minimum visits recommendation for prenatal and postnatal care for women of childbearing age [22]. The utilization of maternal health services is reported to vary within developing countries, with majority of findings showing differences between affluent and poor women, and between women living in urban and rural areas [40, 41]. Shen et al. found the use of maternal health services in western rural China was significantly unequal between pregnant women of poor and non-poor economic statuses [42]. Say and Raine found wealthier women were usually more likely to receive early prenatal visits than poor women in India [1]. Li et al. revealed that richer women gain greater benefit from the publicly provided prenatal health services than poorer women and it was underutilized in rural Shaanxi province, China [43]. One of the encouraging findings in this study is that the new health system reform has significantly diminished the inequality of prenatal visits among urban women, as well as led to the utilizations of postnatal visits more concentrated among the poor urban women. The changes of maternal health services utilizations in rural women were not statistically significant. In addition, it seemed that the qualified prenatal and postnatal visits were increased among rural women post the health system reform after adjusting the potential determinants, which means the new health system reform had improved the frequency of prenatal and postnatal visits among rural women.

Taguchi et al. and Pallikadavath et al. pointed out that higher education level of women was associated with greater use of prenatal visits [44, 45]. Similarly, our results indicated a positive association between women’s education and prenatal visits. Both urban and rural women with a low education level were more likely to receive less prenatal visits when adjusting for the other factors, which was consistent with other investigations in China [46, 47]. A possible interpretation was that more educated women tend to realize the importance of prenatal care during pregnancy. Parity was one significant factor influencing rural women’s prenatal visits; compared to women with one or less parity, rural women with more than two parities were more likely to receive less qualified prenatal visits, which was consistent with what have been reported by Agus et al. and Trinch et al. [31, 32]. One possible explanation was that women may have a relatively weaker realization of the importance of prenatal visits when they have had at least one delivery. A previous study showed poor economic status was a determinant of low uptake of prenatal visits [33], while in this study we found the richest household income of urban women received less qualified prenatal visits. This inconsistent conclusion may be related to the insufficient sample size of this study, with further research needed regarding the association of household income with prenatal visits.

Our results also indicated that the main indictor of receiving more qualified postnatal visits was the primary delivery institution for urban women. When a woman delivers at a primary delivery institution, it is ensured that more health workers are available than at secondary and above institution, and they assess the mother’s situation within a few hours of childbirth [34]. Therefore, the current finding was in line with the guidelines of maternal health services. As for rural women, the striking finding was that women’s education had a strong association with qualified postnatal visits which was similar to the findings by Mohan et al. in rural Tanzania [48]. It may be due to higher educational attainment, which is more often associated with greater awareness of the utilization of maternal health services [49].

There are two limitations to this study that should be considered when interpreting the results. Firstly, all the data were collected by a self-report approach, and there may be recall bias. However, pregnancy and childbirth are events that women remember for years, thus the recall bias could be negligible. Secondly, the determinants of prenatal and postnatal visit to be considered are limited by the pre-specified questions in the survey. There could be some potential unobserved confounding factors we did not control for.

## Conclusions

The results from this study suggested that the status utilization of prenatal and postnatal visits met the requirement of WHO, and were higher than other areas in China and other developing countries post the new health system reform. It is also suggested that the health system reform reduced the inequality of the utilization of prenatal visits in rich urban women, and increased the utilization of postnatal visits in poor urban women. In addition, the health system reform did not reduce the inequality of the maternal health care utilization of prenatal and postnatal visits among rural women, but it increased the frequency of prenatal and postnatal visits of rural women. It is essential to increase the awareness of the importance of prenatal and postnatal visits, especially among low educated women.

## Additional files

Additional file 1: National Health Service Survey Questionnaire in 2008. (DOCX 41 kb)

Additional file 2: National Health Service Survey Questionnaire in 2013. (DOCX 41 kb

## Abbreviations

CIs: Confidence Intervals
MMR: Maternal Mortality Rate
NHSS: National Health Service Survey
OR: Odds Risk
WHO: World Health Organization
