# Supplementary material for: Exploring status and determinants of prenatal and postnatal visits in western China: in the background of the new health system reform
Source: BMC Public Health. 2017 Jul 20;18:39. doi: 10.1186/s12889-017-4601-4 (PMC5520235; doi:10.1186/s12889-017-4601-4)
Supplement: Supplementary file 1 — National Health Service Survey Questionnaire in 2008. (DOCX 41 kb) [file 12889_2017_4601_MOESM1_ESM.docx]

**Household Questionnaire**

**The National Health Service Survey in 2008**

**Health’s ministry of the People’s Republic of China**

**June 2008**

**The Declaration of Household Survey’s investigator**

**Dear residents,**

**Many greetings!**

**We are the investigators from the Fourth National Health Service Survey. This investigation is organized by the National Health Administration Department and is approved by the National Bureau of Statistics. The purpose of the National Health Service Survey is to understand the residents’ healthy status and provide the basic reference to the health policy makers. According to the National statistical law of the People’s Republic of China’s relevant regulations, the whole questions we investigated is promised to be secrets and only be used for statistical analyses. We hope you can answer the following questions honestly. Thank you very much for your support and cooperation!**

| **This survey is a single survey of private families; information shall not be divulged without consent"** |  | **Table No.: National Health Survey 1 Table** |
| --- | --- | --- |
|  |  | **Establishment department: National Health’s ministry** |
| **《National statistical law of The People’s Republic of China》(The third chapter, the fourteenth article)** |  | **Approval authority: National Bureau of Statistics** |
|  |  | **Approval number: National Bureau of Statistics draft [2008]No.18** |

**The Home Page of the Household Questionnaire**

**Name of householder: _______________ telephone number: _______________**

**Family Address: ________County (City / district) ________Town (Street)**

**_______Village (Neighborhood committee) ____________ (Detailed address)**

**County (City / district) Administrative area No.□□□□□□**

**Town (Street) area No. □ Village (Neighborhood committee) area No. □**

**Resident code □□**

**When you have finished your investigation, fill in the following form (the result is filled in the right space)**

| The number of resident within 6 months: |  |
| --- | --- |
| Including: married women aged 15-49 years old |  |
| Including: children aged ≤ 5 years old |  |
| Including: aging people aged ≥ 60 years old |  |
| The number of people who had been injured within the first two weeks of the survey |  |
| The number of people who had been out of hospital within 12 months before the survey |  |
| (In rural area)The number of people who work outside their hometown and their accompanying dependents |  |
| The type of this household: (1) Selected (2) Substituted |  |

**The end date of Investigation: ____________ Investigator (Signature):**

**Verification date: ____________ Investigation Instructor (Signature):**

**Table I Questionnaire of Family’s Basic Characteristics**

**One family member who is familiar with the family’s basic characteristics was asked to answer the questions in table 1**

| No. | Questions and options | Answer |
| --- | --- | --- |
| 1 | How many people are there in your family? (Including the people in your household register, relatives, housekeeper and so on? |  |
| 2. | How many people who are at home during 6 months? |  |
| 3 | (In the rural area) How many people are there working outside the county town?( including their spouse, children, parents and so on) |  |
| 4 | What type of house do you have? (1) Building (2) Brick bungalow (3) Adobe bungalow (4) Others |  |
| 5 | How many square meters is your house? (m^2^) |  |
| 6 | How many televisions do you have? |  |
| 7 | What’s the type of your telephone? (1) Fixed telephone (2) Mobil phone (3) Both of them (4) None |  |
| 8 | What type of drinking water do you have? (1) Running water (2) Hand press well water (3) Protected well water (4) Collected Rainwater (5) Protected spring water (6) Unprotected well water (7) Unprotected spring water (8) Water from truck or trolley (9) Surface water (10) Canned water and the above 1-5 are used for cooking and washing (11) Canned water and the above 6-9 are used for cooking and washing (12)Others |  |
| 9 | What type of washroom do you have? (1) Complete the sewer flushing (2) Urine diversion (3) Triplex methane (4) Funnel type (5) Three compartment septic tank (6) Double pit alternation (7) Ventilation type (8) Attic type (9) Pit antifreeze (10) Pit type toilet with cover plate (11) Pit type toilet without cover plate (12) manure bucket (13) Facility free / field (14) Others |  |
| 10 | How many kilometers is the nearest medical institution from your home?  (1) < 1km (2) 1- (3) 2- (4) 3- (5) 4- (6) ≥ 5 |  |
| 11 | How many minutes does it take from your home to the nearest medical institution?  (The fastest way to get on foot or by transport) |  |
| No. | Questions and options | Answer |
| 12 | How much was the total household income in the last year? (RMB)( Urban households are disposable income, rural households are net income) |  |
| 13 | How much was the total household consumption expenditure in the last year? (RMB) |  |
| 14 | Including: How much was the food expenditure? |  |
| 15 | How much was the daily living expenditure? |  |
| 16 | How much was the transportation and communication expenditure? |  |
| 17 | How much was the housing, water, electricity and fuel expenditure |  |
| 18 | How much was the education expenditure |  |
| 19 | How much was the entertainment expenditure |  |
| 20 | Including: How much was the health care expenditure |  |
| 21 | How much was the Others expenditure? |  |
| 22 | How much is the tuition for children outside their hometown? (RMB) |  |
| 23 | Last year, how much was it equivalent to RMB that your family enjoyed any kind of subsidy from the state or the collective? (Fill in 0 if there is none) |  |
| 24 | Is your home identified as a government target for medical assistance? (1) Yes (2) No (3) I don’t known |  |
| 25 | Is your household listed as a local poverty-stricken or low-income household? (1) Yes (2) No (3) Both of them (4) None |  |
| 26 | If yes, what is the most important reason leading to the poverty-stricken household or low-income household? (1) Small size of labor (2) Poor natural condition/disaster(3) Labor affected by disease (4) disease (5)Unemployment (6) human factor (7) Others |  |

**Table II Questionnaire of Family member’s Basic Characteristics**

| **Investigated members’ code** (01 represent the householder, Other family members are numbered in accordance with the survey order. Once the code is determined, it cannot be changed.) | | 01 | 02 | 03 | 04 | 05 | 06 |
| --- | --- | --- | --- | --- | --- | --- | --- |
| **A. Family member’s basic information** | |  |  |  |  |  |  |
| 1 | Member’s name: (01 is the householder) |  |  |  |  |  |  |
| 2 | The relationship between the member and householder:  (1)The householder (2) Spouse (3)Children (4) Grandchildren (5)Parents (6) Grandparent  (7)Brothers/Sisters(8)Others |  |  |  |  |  |  |
| 3 | Who will answer the following survey questions (**Investigators judge by themselves**)?  (1)By oneself (2) Reply by others |  |  |  |  |  |  |
| 4 | The reason of answering by others: (1) This family member is out (2) This family member is too young (3) This family member is in a insobriety (4) This family member is unwilling to answer (5)Others |  |  |  |  |  |  |
| 5 | Sex: (1) Male (2) Female |  |  |  |  |  |  |
| 6 | Ethnical: (1)Han (2) Mongol (3)Hui (4) Tibet (5) Uygurs (6) Miao (7) Others |  |  |  |  |  |  |
| 7 | Birth Date: Year ________ (fill in four numbers, for example: 1998) |  |  |  |  |  |  |
| 8 | Birth Date: Month ________ |  |  |  |  |  |  |
| **Continue to ask the members aged more than 15 years old** (member who were born before June, 1993)**, the other members skip to question 13** | | | | | | | |
| 9 | Marital status:  (1) Single (2) Married (3) Widowed (4) Divorce (5) Others |  |  |  |  |  |  |
| 10 | Education:  (1) Illiteracy (2) Primary school (3) Middle school (4) High school/ Technical school (5) Secondary specialized school (6) Junior College (7) undergraduate course and above |  |  |  |  |  |  |
| 11 | Employment status:  (1) Employed (2) Retirement (3) Student at school (4)Out of work/Unemployment |  |  |  |  |  |  |
| **Investigated members’ code** | | 01 | 02 | 03 | 04 | 05 | 06 |
| 12 | Type of work: (1) Person in charge of enterprise and institution (2) Professional and technical personnel (3) Administrative staff (4) Business / service personnel (5) Individual industrial and commercial households (6) Non-Agricultural production personnel (7)Non-farm worker (8) Agricultural laborers(Agricultural and forestry, fishery and water conservancy production personnel) (9)Others |  |  |  |  |  |  |
| 13 | What type of medical insurance scheme do you have? (1) Urban Employee Basic Medical Insurance (2) Free medical care (3) Urban Resident Basic Medical Insurance (4) New Cooperative of Rural Medical Insurance (5) Others social medical Insurance (6)Don’t attend |  |  |  |  |  |  |
| 14 | How much do you pay if you attend urban resident basic medical insurance or new cooperative of rural medical insurance? (RMB) |  |  |  |  |  |  |
| 15 | Have you purchased commercial medical insurance? (1) Yes (2) No |  |  |  |  |  |  |
| 16 | How much do you pay if you purchase commercial medical insurance? (RMB) |  |  |  |  |  |  |

**Table III Questionnaire of women aged 15-64 years old**

**The women who were born from August 16^th^ 1949 to August 15^th^ 1998 answer the following questions**

| **Investigated members’ code (the code is the same as table 2)** | | 01 | 02 | 03 | 04 | 05 | 06 |
| --- | --- | --- | --- | --- | --- | --- | --- |
| 1 | Have you had a gynecological examination in the past 12 months (breast, uterus and so on)? (1) Yes (2) No |  |  |  |  |  |  |
| 2 | How many times have you been pregnant? (Including abortion) |  |  |  |  |  |  |
| 3 | How many children have you had? (Including the children that had dead) |  |  |  |  |  |  |
| 4 | Have you had any child who was unfortunately died since 2003?  (1) Yes (2) No (Skip to question 10) |  |  |  |  |  |  |
| 5 | If yes, how many? |  |  |  |  |  |  |
| 6 | The date of the first child who was dead: (1)Jan-Jun 2003 (2)Jul-Dec 2003 (3) 2004  (4) 2005 (5) 2006 (6) 2007 (7) 2008 |  |  |  |  |  |  |
| 7 | The age of the dead child? (1)less than 1 years old (2) 1 years old (3) 2 years old  (4) 3 years old (5) 4 years old (6) more than 5 years old |  |  |  |  |  |  |
| 8 | The date of the second child who was dead: (1)Jan-Jun 2003 (2)Jul-Dec 2003 (3) 2004  (4) 2005 (5) 2006 (6) 2007 (7) 2008 |  |  |  |  |  |  |
| 9 | The age of the dead child?  (1)less than 1 years old (2) 1 years old (3) 2 years old  (4) 3 years old (5) 4 years old (6) more than 5 years old |  |  |  |  |  |  |
| 10 | (In the rural area) Does your husband work long distances outside your hometown?  (1) Yes (2) No |  |  |  |  |  |  |
| **Investigated members’ code** | | 01 | 02 | 03 | 04 | 05 | 06 |
| 11 | (In the rural area)Have you got medical services provided by female doctors when you demand? (1) Yes (2) No (3) Never demand (4) I don’t known |  |  |  |  |  |  |
| 12 | Do you give a birth since Jan 1^st^ 2003?  (1) Yes (2) No (The investigation is end) |  |  |  |  |  |  |
| All of the following questions are asked about the status of the last child and his/her mother during pregnancy | | | | | | | |
| 13 | The last birth date of a child: Year ________ |  |  |  |  |  |  |
| 14 | Month ________ |  |  |  |  |  |  |
| 15 | The sex of your latest child?  (1)Male (2)Female |  |  |  |  |  |  |
| 16 | How many times of prenatal visits have you had? (if one have no prenatal visit, fill in 0 and skip to the question 20) |  |  |  |  |  |  |
| 17 | When did the first time of prenatal visit? (week) |  |  |  |  |  |  |
| 18 | Where did you do the prenatal visits? (not more than 3 options)  (1) County Hospital and above (2) County Chinese Hospital and above (3)Maternal and child health care institution (4)Township Health Center (5) Community Health Centre  (6) Family planning guidance Station (7) Village clinic (8)At home |  |  |  |  |  |  |
| 19 | During the prenatal visit, did the doctor do the following examination? |  |  |  |  |  |  |
| 19.1 | Measure weight: (1) Yes (2) No |  |  |  |  |  |  |
| 19.2 | Do blood test: (1) Yes (2) No |  |  |  |  |  |  |
| 19.3 | Measure blood pressure: (1) Yes (2) No |  |  |  |  |  |  |
| **Investigated members’ code** | | 01 | 02 | 03 | 04 | 05 | 06 |
| 19.4 | Urine test : (1) Yes (2) No |  |  |  |  |  |  |
| 19.5 | B-ultrasonic examination: (1) Yes (2) No |  |  |  |  |  |  |
| 20 | What was the gestational week? (week) |  |  |  |  |  |  |
| 21 | What was the delivery way? (1) Vaginal (2) Vaginal Midwifery (3) Caesarean section |  |  |  |  |  |  |
| 22 | What was the delivery institution?  (1) County Hospital and above (2) County Chinese Hospital and above (3)Maternal and child health care institution (4)Township Health Center (5) Community Health Centre (6) Family planning guidance Station (7) Village clinic (8)At home (9)Others |  |  |  |  |  |  |
| 23 | What was the reason gave birth at home? (1)there is no need to hospital (2) There's not enough time (3)lack of money (4) Inconvenient transportation (5)Others |  |  |  |  |  |  |
| 24 | Who was the midwife if women gave birth at home?(1) Doctor from township and above (2) Village doctor (3) Midwife (4) Part-time delivery personnel (5) Family member (6) Others |  |  |  |  |  |  |
| 25 | What was the weight of newborn? (g) |  |  |  |  |  |  |
| 26 | What was the total cost of delivery? (RMB) |  |  |  |  |  |  |
| 27 | How much was reimbursement? (Fill in 0 If there was no ) |  |  |  |  |  |  |
| 28 | How many times of postnatal visits did you receive during 42 days after delivery? (Fill in 0 If there was no ) |  |  |  |  |  |  |
| 29 | How long did you first breastfeed your baby?  (1)Half an hour after born (2)between half an hour and an hour after born (3) within 24 hours (4)after 24hours (5) Never |  |  |  |  |  |  |
