# Supplementary material for: Exploring status and determinants of prenatal and postnatal visits in western China: in the background of the new health system reform
Source: BMC Public Health. 2017 Jul 20;18:39. doi: 10.1186/s12889-017-4601-4 (PMC5520235; doi:10.1186/s12889-017-4601-4)
Supplement: Supplementary file 2 — National Health Service Survey Questionnaire in 2013. (DOCX 41 kb [file 12889_2017_4601_MOESM2_ESM.docx]

Table No.: National Health Survey 1 Table

Establishment department: National Health and Family Planning Commission

Approval authority: National Bureau of Statistics

Approval number: National Bureau of Statistics draft [2013]No.65

Expiration date: December 2013

**Household Questionnaire-The National Health Service Survey in 2013**

Family Address: _____County(City / district)_____Town(Street)_____Village(Neighborhood committee)_________________(Detailed address)

Name of householder: ____________________ telephone number: _____________________

County(City / district) Administrative area No.□□□□□□ Town(Street) area No. □□□ Village(Neighborhood committee) area No. □□□

Resident code □□

The start date of Investigation: _______________ _____:_____

The end date of Investigation: ________________ _____:_____ Investigator (Signature):

Verification date: _____________ Investigation Instructor (Signature):

**The Declaration of Household Survey’s investigator**

Dear residents,

Many greetings!

We are the investigators from the Fourth National Health Service Survey. This investigation is organized by the National Health Administration Department and is approved by the National Bureau of Statistics. The purpose of the National Health Service Survey is to understand the residents’ healthy status and utilization of health care, to provide the basic reference to the health policy makers and improve the level of residents’ health. According to the National statistical law of The People’s Republic of China’s relevant regulations, the whole personal information we investigated is promised to be secrets and only be used for statistical analyses. We hope you can answer the following questions honestly. Thank you very much for your support and cooperation!

“Anyone or any departments are not allowed to leak out the direct or indirect personal information which were got in statistical investigation to others. This information can only be used for statistical analyses”

《National statistical law of The People’s Republic of China》(The third chapter, the twenty-fifth article)

**The Detailed List of Survey**

Table I Questionnaire of Family’s Basic Characteristics

Table II Questionnaire of Family member’s Basic Characteristics

……

Table VI Questionnaire of women aged 15-64 years old

**Table I Questionnaire of Family’s Basic Characteristics**

**One family member who is familiar with the family’s basic characteristics was asked to answer the questions in table 1**

| No. | Questions and options | Answer |
| --- | --- | --- |
| 1 | How many people are there in the household register? |  |
| 2. | How many people who are in the household register are at home during 6 months? |  |
| 3 | How many people are there at home during 6 months but not in the household register? (including relatives, housekeeper and so on) |  |
| 4 | (In the rural area) How many household register people are there working outside the countytown?( including their spouse, children, parents and so on) |  |
| 5 | How many kilometers is the nearest medical institution from your home? (1) < 1km (2) 1- (3) 2- (4) 3- (5) 4- (6) ≥ 5 |  |
| 6 | How many minutes does it take from your home to the nearest medical institution? (The fastest way to get on foot or by transport) |  |
| 7 | What kind of medical institution do you usually go to for general diseases: (1) village clinic (2) Community Health Station (3) Township Health Center (4) Community Health Centre (5) comprehensive hospital (6) Chinese medicine hospital (7) Others |  |
| 8 | Compared with 5 years age, How has your family changed in the convenience of visiting a doctor? (1) Substantially improved (2) Slight improved (3) unchanged (4) Slightly worse (5) Substantially worse |  |
| 9 | Compared with 5 years age, How has your family changed in the cost of visiting a doctor? (1) Substantially decreased (2) Slight decreased (3) unchanged (4) Slightly increased (5) Substantially increased |  |
| 10 | What kind of relationship do you think doctors and patients are most similar to? (1) Parents and children (2) Teachers and students (3) Friends (4) Work partner (5) Army friends (6) Superior and subordinate (7) Trading services (8) Others |  |
| 11 | What is the most frequently used fuel in your cooking? (1) electricity (2) Coal gas/Natural gas/Petroleum gas (3) Methane gas (4) Coal oil (5) Coal (6) Firewood (7) Others |  |
| 12 | What type of drinking water do you have? (1) Running water (2) Hand press well water (3) Protected well water (4) Collected Rainwater (5) Protected spring water (6) Unprotected well water (7) Unprotected spring water (8) Water from truck or trolley (9) Surface water (10) Others |  |
| 13 | What type of washroom do you have? (1) Complete the sewer flushing (2) Urine diversion (3) Triplex methane (4) Funnel type (5) Three compartment septic tank (6) Double pit alternation (7) Ventilation type (8) Attic type (9) Pit antifreeze (10) Pit type toilet with cover plate (11) Pit type toilet without cover plate (12) manure bucket (13) Facility free / field (14) Others |  |
| 14 | What type of house do you have? (1) Building (2) Brick bungalow (3) Adobe bungalow (4) Others |  |
| 15 | How many square meters is your house? (m^2^) |  |
| 16 | How much was the total household income in the last year? (RMB) (Urban households are disposable income, rural households are net income) |  |
| 17 | How much was the total household consumption expenditure in the last year? (RMB) |  |
| 18 | Including: How much was the food expenditure? |  |
| 19 | How much was the daily living expenditure? |  |
| 20 | How much was the transportation and communication expenditure? |  |
| 21 | How much was the housing, water, electricity and fuel expenditure |  |
| 22 | How much was the education expenditure |  |
| 23 | How much was the entertainment expenditure |  |
| 24 | Including: How much was the health care expenditure |  |
| 25 | How much was the Others expenditure? |  |
| 26 | Is your household listed as a local poverty-stricken household? (1) Yes (2) No |  |
| 27 | Is your household listed as a local low-income household? (1) Yes (2) No |  |
| 28 | What is the most important reason leading to the poverty-stricken household or low-income household? (1) Small size of labor (2) Poor natural condition/disaster(3) Labor affected by disease (4) disease (5)Unemployment (6) human factor (7) Others |  |

**Table II Questionnaire of Family member’s Basic Characteristics**

| **A. Family member’s basic information** | | | | | | | |
| --- | --- | --- | --- | --- | --- | --- | --- |
| **Investigated members’ code** (01 represent the householder, Other family members are numbered in accordance with the survey order. Once the code is determined, it cannot be changed.) | | 01  (householder) | 02 | 03 | 04 | 05 | 06 |
| 29 | Member’s name: (01 is the householder) |  |  |  |  |  |  |
| 30 | The relationship between the member and householder:  (1)The householder (2) Spouse (3)Children (4) Son-in-law/ Daughter-in-law (5)Parents  (6) Wife's parents/Husband's parents (7) Grandparent (8) Grandchildren (9)Brothers/Sisters  (10) Domestic service personnel (11)Others |  |  |  |  |  |  |
| 31 | Who will answer the following survey questions (**Investigators judge by themselves**)?  (1)By oneself (2) Reply by others |  |  |  |  |  |  |
| 32 | household registration place: (1) County/District (2) Other County/District (3) Other province (4) Undetermined household registration place |  |  |  |  |  |  |
| 33 | The type of household registration: (1) Agriculture (2) None-agriculture |  |  |  |  |  |  |
| 34 | Sex: (1) Male (2) Female |  |  |  |  |  |  |
| 35 | Ethnical: (1)Han (2)Zhuang (3)Hui (4) Uygurs (5) Mongol (6)Tibet (7) Manchu  (8) Miao (9)Others |  |  |  |  |  |  |
| 36 | Birth Date: Year ________ (fill in four numbers, for example: 1998) |  |  |  |  |  |  |
| 37 | Birth Date: Month ________ (fill in two numbers, for example: 07) |  |  |  |  |  |  |
| 38 | What is your height? (cm) |  |  |  |  |  |  |
| **Investigated members’ code** | | 01 | 02 | 03 | 04 | 05 | 06 |
| 39 | What is your weight? (kg) |  |  |  |  |  |  |
| 40 | Have you attended the Urban Employee Basic Medical Insurance scheme? (1) Yes (2) No |  |  |  |  |  |  |
| 41 | Have you attended the Urban Resident Basic Medical Insurance scheme? (1) Yes (2) No |  |  |  |  |  |  |
| 42 | Have you attended the New Cooperative of Rural Medical Insurance scheme? (1) Yes (2) No |  |  |  |  |  |  |
| 43 | Have you attended the cooperative medical insurance for urban and rural residents?  (1) Yes (2) No |  |  |  |  |  |  |
| 44 | Have you purchased commercial medical insurance? (1) Yes (2) No |  |  |  |  |  |  |
| 45 | Have you attended the other medical insurance? (1) Yes (2) No |  |  |  |  |  |  |
| 46 | Are you the government's target of medical aid? (1) Yes (2) No (3) I don’t known |  |  |  |  |  |  |
| **Continue to ask the members aged more than 15 years old** (member who were born before August 15^th^, 1998)**, the other members skip to Table 3** | | | | | | | |
| 47 | Marital status: (1) Single (2) Married (3) Widowed (4) Divorce (5) Others |  |  |  |  |  |  |
| 48 | Education:  (1) Illiteracy (2) Primary school (3) Middle school (4) High school (5) Technical school (6) Secondary specialized school:(7) Junior College (8) undergraduate course and above |  |  |  |  |  |  |
| 49 | Employment status:  (1) Employed (2) Retirement (3) Student at school (4)Out of work (5) Unemployment |  |  |  |  |  |  |
| 50 | Type of work: (1) Person in charge of enterprise and institution (2) Professional and technical personnel (3) Administrative staff (4) Business / service personnel (5) Agricultural and forestry, fishery and water conservancy production personnel (6)Operators of production and transportation equipment (7) Soldier (8)Others |  |  |  |  |  |  |

**Table VI Questionnaire of women aged 15-64 years old**

**The women who were born from August 16^th^ 1949 to August 15^th^ 1998 answer the following questions**

| **Investigated members’ code** | | 01 | 02 | 03 | 04 | 05 | 06 |
| --- | --- | --- | --- | --- | --- | --- | --- |
| 167 | Have you had a gynecological examination in the past 12 months? (1) Yes (2) No |  |  |  |  |  |  |
| 168 | Have you had a smear test in the past 12 months? (1) Yes (2) No |  |  |  |  |  |  |
| 169 | Have you had a breast examination in the past 12 months? (1) Yes (2) No |  |  |  |  |  |  |
| 170 | (In the rural area)Have you got medical services provided by female doctors when you demand? (1) Yes (2) No (3) Never demand (4) I don’t known |  |  |  |  |  |  |
| 171 | (In the rural area) Does your husband work long distances outside your hometown?  (1) Yes (2) No |  |  |  |  |  |  |
| 172 | How many times have you been pregnant? (If women have never been pregnant, fill in 0 and stop this investigation) |  |  |  |  |  |  |
| 173 | How many children have you had? (If women have never a child, fill in 0 and stop this investigation) |  |  |  |  |  |  |
| 174 | The latest delivery date: Year ________ (fill in four numbers, for example: 1998) |  |  |  |  |  |  |
| 175 | Month ________ (fill in two numbers, for example: 07) |  |  |  |  |  |  |
| **Women who gave a birth since August 15^th^ 2008 answer the following questions** | | | | | | | |
| 176 | The sex of your latest child?  (1)Male (2)Female |  |  |  |  |  |  |
| 177 | How many times of prenatal visits have you had? (if one have no prenatal visit, fill in 0 and skip to the next question) |  |  |  |  |  |  |
| **Investigated members’ code** | | 01 | 02 | 03 | 04 | 05 | 06 |
| 178 | Did you have a blood test during the prenatal visits? (1) Yes (2) No |  |  |  |  |  |  |
| 179 | Did you measure your blood pressure during the prenatal visits? (1) Yes (2) No |  |  |  |  |  |  |
| 180 | Did you have a urine test during the prenatal visits? (1) Yes (2) No |  |  |  |  |  |  |
| 181 | Did you have a B-ultrasonic examination during the prenatal visits? (1) Yes (2) No |  |  |  |  |  |  |
| 182 | What was the delivery way? (1) Vaginal (2) Caesarean |  |  |  |  |  |  |
| 183 | Who proposed the caesarean section? |  |  |  |  |  |  |
| 184 | What was the delivery institution?  (1) County Hospital and above (2) Maternal and child health care institution (3) Township Health Center (4) Community Health Centre (5) Village clinic (6) At home |  |  |  |  |  |  |
| 185 | Who was the midwife if women gave birth at home?  (1) Doctor from township and above (2) Village doctor (3) Midwife (4) Part-time delivery personnel (5) Family member (6) Others |  |  |  |  |  |  |
| 186 | What was the weight of newborn? (g) |  |  |  |  |  |  |
| 187 | What was the total cost of delivery? (RMB) |  |  |  |  |  |  |
| 188 | How much did you pay for it? (Fill in 0 If there was no )  (Expenses from reimbursement and personal medical accounts are excluded) |  |  |  |  |  |  |
| 189 | How many times of postnatal visits did you receive during 42 days after delivery? (Fill in 0 If there was no ) |  |  |  |  |  |  |

**When you have finished your investigation, fill in the following form (the result is filled in the right space)**

| 1 | The number of resident within 6 months: |  |
| --- | --- | --- |
| 2 | Including: women aged 15-49 years old |  |
| 3 | Including: children aged ≤ 5 years old |  |
| 4 | The number of people who had been injured within the first two weeks of the survey |  |
| 5 | The number of people who had been out of hospital within 12 months before the survey |  |
| 6 | The type of this household: (1) The first investigation (2) The second investigation |  |
| 7 | The type of this household: (1) Selected (2) Substituted |  |
| 8 | The duration of investigation (minutes): |  |
